# Griscelli Syndrome With Hemophagocytic Lymphohistiocytosis: A Rare Case Report

**DOI:** 10.7759/cureus.44445

**Published:** 2023-08-31

**Authors:** Ratna Krishna Tanmayi Perugu, Nanditha Karra, Saniya s Shaik, Womesh Chandra Venigalla, Preeti G, Manasvi Reddy Maram

**Affiliations:** 1 Pediatric Medicine, Osmania Medical College, Hyderabad, IND; 2 Pediatric Medicine, Niloufer Hospital, Hyderabad, IND

**Keywords:** pediatrics, genetics, hematopoietic stem cell transplantation, primary immunodeficiency, silvery grey hair, hypopigmentation, rab27a gene, recurrent infections, hemophagocytic lymphohistiocytosis, griscelli syndrome

## Abstract

Griscelli syndrome type 2 (GS2) is a rare, autosomal recessive condition caused by a mutation of the RAB27A gene that causes primary immunodeficiency and pigmentary dilution of skin and hair. It is a rare occurrence, with only 160 cases reported all over the world. It commonly progresses to hemophagocytic lymphohistiocytosis (HLH) due to immunodeficiency. We herein represent the case of a seven-month-old male child, the firstborn of a third-degree consanguineous marriage, who presented with recurrent viral infections and silvery grey hair. A definitive diagnosis of GS 2 was made in accordance with the pathognomonic appearance of hair on microscopic examination and whole genome sequencing, which revealed a homozygous missense mutation in exon 3 of the RAB27A gene. This article is being reported to highlight the rare incidence of this disease, its overlapping clinical features with malnutrition, the challenges faced in diagnosis, and the treatment modalities for it.

## Introduction

Griscelli syndrome (GS), named after the scientist who first described it, is a rare genetic autosomal recessive condition affecting intracellular vesicular trafficking mechanisms. It is primarily characterized by hypomelanosis - pale skin and silvery grey hair, which is due to defective transport of melanosomes in the melanocytes. Depending on the gene affected, it may be associated with either neurological or immunological manifestations. GS type 1 results from the mutation of the MYO5A gene, which is also involved in axonal transport, thereby giving rise to concurrent neurological abnormalities. GS type 2 (GS2) occurs due to mutations of the RAB27A gene [1], resulting in impaired lymphocyte and natural killer cell function. The inability to clear off pathogens causes chronic overstimulation of T-lymphocytes and macrophages, leading to their uncontrolled activation and proliferation, which is often precipitated by a viral infection. The disease then takes on an accelerated course called hemophagocytic lymphohistiocytosis (HLH), which is marked by splenomegaly, fever, cytopenias of different cell lineages, increased serum triglycerides, increased serum ferritin, and decreased serum fibrinogen [1]. The only potential cure available for GS2 is hematopoietic stem cell transplantation (HSCT) [1], which may yield a good outcome when undertaken promptly. Without treatment, the condition is fatal. GS 3 is due to mutations in the MLPH gene that codes for melanophilin. It presents with isolated pigmentary dilution features without any neurological or immunological manifestations.

Described below is a case of type 2 Griscelli syndrome, a syndrome of rare occurrence with only a total of about 160 cases reported the world over [1].

## Case presentation

A seven-month-old boy was brought to the hospital by his mother with complaints of a low-grade fever associated with intermittent mottling of the skin and no other complaints. He had a past history of measles one month ago and varicella illness 15 days ago, which were managed appropriately. There is no history of seizure episodes. The baby is the firstborn child of a third-degree consanguineous marriage and a product of natural conception. The mother had previously suffered a spontaneous miscarriage at two months of gestational age. The antenatal and perinatal periods of the present baby were uneventful; the baby was born via normal vaginal delivery at full term, and the birth weight was 3.2 kg. All the developmental milestones are normal as per age; the baby is immunized appropriately for age; there is no significant family history.

**Figure 1:**
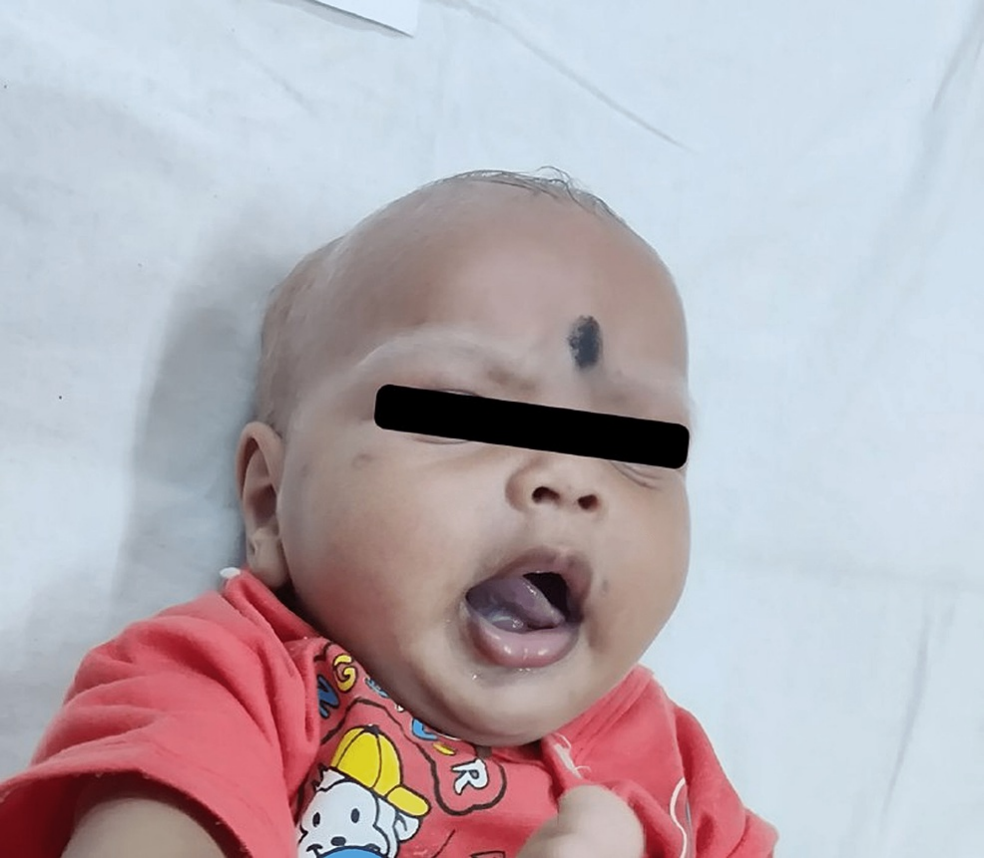
Picture of the baby showing silvery grey scalp hair and eyebrows

On examination, the baby was conscious and active; there were no gross facial dysmorphias or skeletal deformities, and anthropometric measurements were within normal limits. Bilateral testes descend into the scrotum. He is fair-skinned, and his scalp hair, eyebrows, and eyelashes are silvery grey in colour (Figure 1). His bilateral iris is dark brown in colour. The tone of the muscles is normal; they are able to move all four limbs against gravity; deep tendon reflexes are normal; stroking the sole elicits an extensor response. The abdomen was soft, the liver was palpable 3 cm below the right costal margin, and the spleen was tender and palpable 2 cm below the left costal margin. All other examination findings were within normal limits.

The hemogram revealed a normocytic, hypochromic picture, decreased haemoglobin levels (6.6 g%), decreased hematocrit (25.3%), and thrombocytopenia (70,000/µL). Differential leucocyte count (Table 1) showed a significant decrease in neutrophils (13%) and an increase in lymphocytes (80%). There were no giant neutrophilic granules on the peripheral blood smear. Lymphocyte subset analysis revealed a low CD3+ and CD4+ cell count (Table 2). Serum IgA and IgM levels were reduced, while serum IgG levels were within normal limits (Table 3). The hair microscopy report showed an uneven distribution of melanin in the hair shaft and the presence of pigment clumps. On whole genome sequencing, a homozygous missense mutation in exon 3 of the RAB27A gene, resulting in the amino acid substitution of isoleucine for threonine at codon 23, was found, consistent with Griscelli syndrome type 2. The bone marrow aspirate did not show any evidence of hemophagocytosis. Serum triglycerides were elevated to 472 mg/dL, and serum ferritin levels were raised to 2540 ng/ml. Serum fibrinogen levels were within the normal range (180 mg/dl). Based on the history, clinical examination, laboratory investigations, hair microscopy report, and confirmation by exome sequencing, a conclusive diagnosis of type 2 Griscelli syndrome with HLH was established. The child was advised about bone marrow transplantation at one year of age. Until then, the child is being monitored for infections and is to be symptomatically reviewed. For his presenting illness, he was treated symptomatically and discharged with multivitamin supplementation.

**Table 1:** Differential leucocyte count

	Result	Normal range
Neutrophils	13%	40–80%
Lymphocytes	80%	20–40%
Monocytes	7%	2–10%
Eosinophils	-	1–6%
Basophils	-	0–1%

**Table 2:** Lymphocyte subset analysis

Lymphocyte subset	Absolute count (cells/µl)	Percentage	Reference ranges
CD3+ (T cells)	3154	44.4%	51–77%
CD4+ (T helper cells)	1359	19.1%	35–56%
CD8+ (T Suppressor cells)	1717	24.2%	12–35%
CD4/CD8 ratio	0.79		1–4
CD19+ (B cells)	2290	33.6%	11–41%
CD16+ CD56+ (NK cells)	1152	16.9%	3–14%

**Table 3:** Serum immunoglobulin

	Results (mg/dl)	Reference range (mg/dl)
Serum IgA	5	70–400
Serum IgM	21	40–230
Serum IgG	709	700–1600

## Discussion

Griscelli syndrome is a rare autosomal recessive disorder that was first reported by Griscelli et al. in 1978 [2]. To date, 160 cases of this disease have been described in the literature, mostly from the Mediterranean and Turkish regions [1]. The mean age for presentation is 17.5 months, with no sex preponderance [3].

Griscelli syndrome is branched into three subtypes based on clinical and genetic differences. Partial albinism of the skin and silvery grey hair are the distinctive features of all three subtypes. GS-1 has predominant neurological dysfunction with normal functioning of the immune system and is due to mutations in the MYO5A gene. GS-2 is the most common type, with 13 cases reported in the Indian literature [1]. It is due to the RAB27A gene mutation. This gene encodes a GTPase, which plays a role in the exocytosis of cell granules in T-cells and NK cells [1,4]. Mutations in this gene cause combined T-B cell immunodeficiency with defects in NK cell activity, leading to primary immunodeficiency and recurrent infections, along with the failure of peripheral transport of melanosomes [5]. This leads to their clumping within the melanocytes of skin and hair, which manifests as partial albinism. GS-2 may progress to HLH [1,6], which is characterized by overwhelming T-cell and macrophage activation and has a poor prognosis unless prompt intervention is initiated. There is no neurological involvement in GS2. GS-3 [7], the least common type, has no nervous or immune system involvement. Its clinical presentation is restricted to hypopigmentation of skin and hair due to a mutation in the MLPH gene. Interventional strategies also vary depending on the subtype; GS-1 and GS-3 have no specific treatments, and only supportive therapies are provided. For GS-2, bone marrow transplantation is the only available curative treatment [1,8,9]. Other modalities, like immunosuppressive therapy, are available but have a poorer prognosis. Early recognition and treatment are good prognostic factors for GS-2.

In this case, the presenting illness, past recurrent infections (measles and varicella), hypopigmented hair, and hepatosplenomegaly initially led to suspicion of malnutrition, but since the anthropometric measurements were within the normal range, it was ruled out. Based on the silvery grey scalp hair, eyebrows, eyelashes, and distorted differential leucocyte counts, along with the history of consanguinity, we considered the differential diagnosis of GS-2 and Chediak-Higashi syndrome. The absence of giant leukocyte granules on the blood smear [10] pointed to GS-2, and genome sequencing was done. It revealed a mutation in exon 3 of the RAB27A gene, confirming the diagnosis.

Since the patient had a molecular diagnosis consistent with HLH, we concluded the case as Griscelli syndrome-2 with HLH based on the criteria laid down by the Histiocyte Society [11,12]. Hepatosplenomegaly, bicytopenia, hypertriglyceridemia, and hyperferritinemia are other features of HLH. The child was advised to have a bone marrow transplant at one year of age, until when he will be monitored regularly.

India ranks third in the world for malnutrition [13]. The constellation of hypopigmented hair and recurrent infections often presents a diagnostic challenge in a developing country like India, as they are frequently associated with malnutrition. Diseases like Griscelli syndrome-2 benefit from early diagnosis and treatment. Overlapping of its distinctive symptoms with malnutrition and a lack of awareness about the disease hinder its early diagnosis. Hematopoietic stem cell transplantation is the curative treatment for GS-2. Through this case report, we aim to highlight the clinical presentation of the disease along with its diagnostic and treatment modalities to increase awareness, facilitate early diagnosis, and prompt intervention.

## Conclusions

Early diagnosis and intervention can help improve the chances of survival and quality of life in an otherwise fatal type 2 Griscelli syndrome. The diagnostic modalities that can be used to diagnose a case of GS2 include hair microscopy, skin biopsy, and gene sequencing. Degranulation assays and serum immunoglobulin levels could serve as corroborating evidence. A hemogram, serum triglyceride levels, serum fibrinogen level, serum ferritin, NK cell activity, and bone marrow aspiration cytology can be used to assess the HLH status of the patient. The potential curative option is hematopoietic stem cell transplantation, with other modalities like immunosuppressive therapy being available but associated with a poorer prognosis. The parents should be counselled regarding the availability of antenatal diagnosis for future pregnancies through chorionic villus sampling.
